# Complete Genome Sequence of Yellow Pigment-Producing *Sphingobium* sp. Strain HAL-16

**DOI:** 10.1128/MRA.00985-20

**Published:** 2020-10-08

**Authors:** Soon Jae Kwon, Pyung Cheon Lee

**Affiliations:** a Department of Molecular Science and Technology, Ajou University, Suwon, South Korea; b Department of Applied Chemistry and Biological Engineering, Ajou University, Suwon, South Korea; Portland State University

## Abstract

*Sphingobium* sp. strain HAL-16, which was isolated from Antarctic soil samples, synthesizes a yellow pigment. The complete genome consists of a single circular chromosome (4,372,398 bp, with a G+C content of 62.7%) and a single circular plasmid (57,025 bp, with a G+C content of 59.4%). Five genes encoding carotenogenic enzymes were identified, suggesting that the yellow pigment is a hydroxy/keto-β-carotene.

## ANNOUNCEMENT

Recently, a deep-yellow *Sphingobium* sp. strain (named HAL-16) was isolated from Antarctic surface soil samples obtained near King Sejong Station on King George Island (62°14′45.4ʺS, 58°46′36.2ʺW). The *Sphingobium* sp. strain HAL-16 produces a yellow pigment, which might be a carotenoid. Carotenoids are widely used as cosmetic ingredients, antioxidants, and food or feed additives (1–3).

The strain was isolated by picking single yellow colonies from lysogeny broth (LB) (BD Difco, USA) agar plates that had been incubated at 25°C and purified by restreaking twice. The strain was aerobically grown in LB at 25°C for 36 h. Genomic DNA was isolated and purified using a genomic DNA extraction kit (Macrogen, South Korea). The genomic DNA was sequenced in single-molecule real-time (SMRT) cells with Pacific Biosciences (PacBio) RS II SMRT technology and on the HiSeq 2000 platform (Illumina, USA). Both types of sequencing were performed by DNA Link, Inc. (Seoul, South Korea). Sequencing libraries were prepared using the SMRTbell template preparation kit v1.0 for PacBio RS II sequencing and the TruSeq Nano DNA kit for Illumina sequencing. After subread filtering of PacBio raw data (read quality, ≥0.75), 239,208 long reads with an average length of 8,157 bp and an *N*_50_ of 61,188 bp (1,951,305,190 bp total, with >440-fold genome coverage) were generated and *de novo* assembled in the Canu v1.3 assembler (4) with the “genomeSize=5m” setting. Overlapping regions at both ends of one contig were trimmed to create unique stretches at both ends using Circlator (5) with the parameter “b2r_length_cutoff= 30000, 60000, 100000 or 200000.” The two resulting assemblies were error corrected via Quiver in SMRT Analysis v2.3.0 for three cycles (6). The corrected assemblies were next polished in Pilon v1.22 (7) (with the parameter fix bases) with trimmed paired-end Illumina reads (3,167,243 reads totaling 639,783,086 bp, with >144-fold genome coverage) that had been obtained from 2 × 101-bp paired-end reads (5,881,775 reads totaling 1,188,118,550 bp) using Sickle v1.33 (https://github.com/najoshi/sickle). The assembly statistics were obtained using the stats.sh script from BBMap v38.68 (https://sourceforge.net/projects/bbmap). Genome annotation was performed via the Prokaryotic Genome Annotation Pipeline (PGAP) (8). CRISPRCasFinder v4.2.20 (9) was utilized for CRISPR/Cas analysis.

The genome of *Sphingobium* sp. strain HAL-16 comprises a single 4,372,398-bp circular chromosome (with a G+C content of 62.7%) and a single 57,025-bp circular plasmid (with a G+C content of 59.4%). Annotation revealed 4,199 coding DNA sequences and 64 RNA genes (3 copies of rRNAs and 55 tRNAs); 16S rRNA gene sequence analysis revealed that the strain HAL-16 sequence has 97.08% similarity to that of Sphingobium scionense WP01^T^. Five genes for hydroxy/keto-β-carotene biosynthesis were predicted in the genome, i.e., one encoding phytoene synthase (CrtB), one encoding phytoene desaturase (CrtI), one encoding lycopene β-cyclase (CrtY), one encoding β-carotene hydroxylase (CrtZ), and two encoding β-carotene ketolase (CrtO). The presence of the five carotenogenic genes strongly supports the idea that the yellow pigment may be a polar carotenoid.

### Data availability.

This project was deposited in DDBJ/EMBL/GenBank under the accession numbers CP060388 (chromosome) and CP060389 (plasmid_HAL16). The SRA/DRA/ERA accession numbers are SRR11775091 (PacBio) and SRR11775090 (Illumina). The BioSample and BioProject numbers are SAMN14846973 and PRJNA630860, respectively.
